# *Mycoplasma pneumoniae* Large DNA Repetitive Elements RepMP1 Show Type Specific Organization among Strains

**DOI:** 10.1371/journal.pone.0047625

**Published:** 2012-10-16

**Authors:** Oxana Musatovova, T. R. Kannan, Joel B. Baseman

**Affiliations:** Department of Microbiology and Immunology, The University of Texas Health Science Center at San Antonio, San Antonio, Texas, United States of America; Monash University, Australia

## Abstract

*Mycoplasma pneumoniae* is the smallest self-replicating bacterium with a streamlined genome of 0.81 Mb. Complete genome analysis revealed the presence of multiple copies of four large repetitive elements (designated RepMP1, RepMP2/3, RepMP4 and RepMP5) that are implicated in creating sequence variations among individual strains. Recently, we described RepMP1-associated sequence variations between reference strain M129 and clinical isolate S1 that involved three RepMP1-genes (i.e. *mpn130*, *mpn137* and *mpn138*). Using PCR and sequencing we analyze 28 additional *M. pneumoniae* strains and demonstrate the existence of S1-like sequence variants in nine strains and M129-like variants in the remaining nineteen strains. We propose a series of recombination steps that facilitates transition from M129- to S1-like sequence variants. Next we examined the remaining RepMP1-genes and observed no other rearrangements related to the repeat element. The only other detected difference was varying numbers of the 21-nucleotide tandem repeats within *mpn127*, *mpn137*, *mpn501* and *mpn524*. Furthermore, typing of strains through analysis of large RepMPs localized within the adhesin P1 operon revealed that sequence divergence involving RepMP1-genes *mpn130*, *mpn137* and *mpn138* is strictly type-specific. Once more our analysis confirmed existence of two highly conserved groups of *M. pneumoniae* strains.

## Introduction

*Mycoplasma pneumoniae* is the causative agent of primary atypical pneumonia and is also associated with a spectrum of other respiratory tract infections, including tracheobronchitis, bronchiolitis, pharyngitis, and croup in children and young adults [1]. Many studies have implicated *M. pneumoniae* in the initiation and persistence of asthma [2]. Furthermore, *M. pneumoniae* infections can lead to gastrointestinal, hematologic, neurological, dermatologic, musculoskeletal, joint, and cardiovascular pathologies, in part manifested as arthritis, pericarditis, and central nervous system disorders [3].

*M. pneumoniae* is among the smallest self-replicating microorganisms with a streamlined genome containing 688 protein-coding genes [4]. Despite its size, 8% of the *M. pneumoniae* genome consists of multiple copies of four large repetitive sequence elements, designated RepMPs (short review of RepMP1-RepMP5 is provided in Table 1) [4], [5], [6], [7]. Preservation of these repetitive sequences during presumed mycoplasma genome minimalization reinforces their importance, as they form pools of sequences for homologous recombinations that yield antigenic variations of *M. pneumoniae* proteins (adhesins and adherence-related proteins, structural components, etc.) Three of four large repeat elements (Table 1) are found within *mpn141*, which encodes the major adhesin protein P1 and *mpn142*, which encodes a precursor processed to adherence-related proteins P90 and P40. P1 gene (*mpn141*) contains one copy of each RepMP2/3 and RepMP4, whereas *mpn142* contains one copy of RepMP5 [7]. Based upon variation in the P1-RepMPs sequence, all worldwide clinical isolates of *M. pneumoniae* can be classified into two distinct, highly conserved groups or types [8], [9]. Prevalence of these groups appears to shift in subsequent epidemic peaks [10], [11]. It was also shown that *mpn142*-RepMP5 sequence variants are type-specific [12], [13]. Despite the existence of two highly conserved groups of P1 genes, sequence variability has been observed within each *M. pneumoniae* type. It is believed that multiple copies of identical or nearly identical RepMP2/3, RepMP4 and RepMP5 sequences located outside *mpn141* and *mpn142* play a part in generation of observed sequence variations [4], [7].

**Table 1:** *M. pneumoniae* large repetitive elements.

Repeat	Repeat size (kb)	Number of copies	Description
RepMP1^a^	0.3	≤20^b^	The only *M. pneumoniae*-specific repeat, RepMP1 possesses the mosaic pattern structure: core element (∼300 bp), accompanied by short repeats, sReps [17]. Currently, RepMP1-proteins have no function assigned, although synthesis of the majority of these proteins has been demonstrated. RepMP1-proteins belong to the largest *M. pneumoniae* family of proteins that is united by the presence of coiled-coil domain (DUF16) within their distal regions [25] .
RepMP2/3^c^	1.8	10	A single copy of each RepMP2/3 and RepMP4 is localized within the cytadhesin P1 gene [21] and multiple additional copies are spread throughout the *M. pneumoniae* chromosome. P1 gene sequence variability, presumably resulting from inter- and intragenic recombination mediated by these repeats, allows classification of clinical *M. pneumoniae* strains into two distinctive and highly conserved types (groups) designated type 1 and 2 [19].
RepMP4^c^	1.1–1.5	8	
RepMP5^c^	1.9–2.2	9	One copy is found within MPN142 coding region that encodes cytadherence-related protein(s) [21], [22] and numerous copies are spread throughout the chromosome. Comparison of different strains revealed that RepMP5-sequence variability is type-specific (RepMP5-1 and RepMP5-2) [12].

a RepMP1 repeat was originally identified by Wenzel and Herrmann [6].

b BLAST analysis of M129 genome revealed twenty full and partial copies of core element as compared to fourteen sequences identified previously.

c Ruland et al. [7] performed analysis of three different repetitive elements contained within P1 operon that yielded both sizes and number of copies spread throughout the M129 chromosome.

Large repetitive elements with homology to RepMP2/3, RepMP4 and RepMP5 are also found in *Mycoplasma gallisepticum* and *Mycoplasma genitalium*. Recently, the large repeats found within *mgpA* and *p110* of *M. genitalium* (homologs of *mpn141* and *mpn142*) were confirmed to be involved in reciprocal intra- and intergenic exchange-mediated variability of encoded adhesin MgpA and protein P110, respectively [14], [15], [16].

In contrast, the large repeat designated RepMP1 is *M. pneumoniae*-specific, and no similar sequences have been identified in any other sequenced mycoplasmas. Numerous copies of RepMP1 were identified in the genome of *M. pneumoniae* strain M129 (Table 1) and genes containing this sequence were named RepMP1-genes [4]. The unique mosaic structure of RepMP1 consists of the ∼300-bp core element and three associated short repeats (designated sRepA, sRepB and sRepC). Individual RepMP1-genes exhibit different combinations of short repeats and core element [17]. RepMP1 has been proposed to create sequence variations through homologous recombination [6], [17]. Recently, we described sequence variations between two *M. pneumoniae* isolates that involved RepMP1 repeats. Comparison of reference strain M129 with clinical isolate S1 showed significant rearrangements in three RepMP1-containing genes, leading to the loss of one coding region (*mpn130*) and fusion of two RepMP1-genes (*mpn137* and *mpn138*) in S1 [5].

Here we analyze RepMP1-genes in 28 additional *M. pneumoniae* strains and observe identical sequence variation in nine strains. We demonstrate that sequence variation involving MPN130, MPN137 and MPN138 is strictly type-specific and propose a model for RepMP1-mediated recombination leading to this divergence. Additionally, in four RepMP1-genes we detect the deletion or insertion of 21-nucleotide tandem repeats within regions that encode a coiled-coil domain of the RepMP1-proteins.

## Materials and Methods

### Cells and chromosomal DNA isolation

*M. pneumoniae* cells were grown to mid-log phase in SP4 medium as previously described [18] and chromosomal DNA was isolated using Easy DNA kit (Invitrogen Corp., Grand Island, NY). and quantified by optical density at 260 nm.

### PCR-RFLP typing of P1 gene (MPN141)

For the PCR-RFLP assay, adhesin P1 genes from all strains were amplified in two products as described earlier [19]. Amplicon ADH1-2 (using primers ADH1: 5′-CTGCCTTGTCCAAGT CCACT-3′ and ADH2: 5′-AACCTTGTCGGGAAGAGCTG-3′) encompassed the proximal half and ADH3-4 (using primers ADH3: 5′-CGAGTTTGCTGCTAACGAGT-3′ and ADH4: 5′-CTTGACTGATACCTGTGCGG-3′) encompassed the distal half of the P1 gene. All PCR amplifications were carried out using Platinum® *Taq* DNA Polymerase High Fidelity kit (Invitrogen Corp., Grand Island, NY) and 30 cycles with 30 s at 94°C, 30 s at 55°C and 3 min at 68°C. Amplified products were subjected to restriction by *Hha*I, *Hpa*II, *Mbo*I, and *Rsa*I (New England BioLabs Inc., Ipswich, MA), and generated restriction patterns were resolved on 2% GenePure agarose (ISC BioExpress, Kaysville, UT) for comparison and analysis.

### Amplification of MPN142 repetitive region for analysis

Additionally, PCR amplification was performed using primers RepMP5F and RepMP5R (Table S1). Primer RepMP5F is complementary to primer ADH4, and reverse primer RepMP5R binds to the MPN142 sequence downstream of the RepMP5 sequence. Again, amplifications were carried out using Platinum® *Taq* DNA Polymerase High Fidelity kit and 30 cycles with 30 s at 94°C, 30 s at 57°C and 2 min at 68°C. Generated products were separated on 1% agarose and their sizes evaluated. To assess sequence differences among strains, amplified regions were restricted by *Hha*I, *Hpa*II, *Mbo*I, and *Rsa*I, and restriction fragments were resolved on 2% GenePure agarose for comparison with patterns generated for strains M129 and FH.

### PCR amplification of *M. pneumoniae* RepMP1-genes

Primers used for PCR amplifications are listed in Table S1. All amplifications were carried on using Platinum® *Taq* DNA Polymerase High Fidelity kit. Amplified products were visualized on 1.5% agarose and sizes were estimated. Prior to sequencing, amplicons were purified using QIAquick® Gel Extraction Kit (QIAGEN, Valencia, CA).

### Sequencing and analysis of the amplified regions

Sequencing was done by the Department of Microbiology and Immunology Nucleic Acids Facility (University of Texas Health Science Center at San Antonio).

Chromosomal region containing *mpn127* of two reference strains maintained in our laboratory (B9-M129 and FH) and S1 clinical strains were sequenced and sequences are provided in Supporting Information (Figure S3, S4 and S5).

Both DNA and deduced amino acid sequences were analyzed using the Basic Local Alignment Search Tool (BLAST) available at the National Center for Biotechnology Information (NCBI) page (http://blast.jcvi.org/cmr-blast/). Alignment of sequences was performed using CLUSTALW. Tandem repeats were identified using software at http://tandem.bu.edu/trf/trf.html. Secondary structure of putative RepMP1-proteins was assessed using ExPASy Proteomics Server (http://expasy.org/tools/#proteome); prediction of trans-membrane regions in prokaryotes was done using the Dense Alignment Surface method (http://www.sbc.su.se/~miklos/DAS/); and prediction of coiled-coil regions was done using COILS (http://www.ch.embnet.org/software/COILS_form.html).

### Nomenclature

Genomic sequence and coding region annotation of *M. pneumoniae* isolate M129 (U00089) were used for identification of individual genes throughout this study. Adhesin P1 [20] is encoded by P1 gene (locus MPN141, nucleotides 180858 to 185741) that is also known as ORF5 [21]. The adjacent downstream coding region (locus MPN142, nucleotides 185747 to 189403), also known as ORF6 [21], encodes cytadherence-related protein(s) [22].

Recently, complete sequences of two additional *M. pneumoniae* strains, FH [CP002077, [23]] and 309 [AP012303, [24]] have become available and were also used for analyses.

The partial sequence generated for Texas strain S1 has been deposited in GenBank by us (EF470909). This sequenced region corresponds to nucleotides 166792 to 187584 in *M. pneumoniae* M129 complete genome, and matches region 166767 to 185839 of strain FH and nucleotides 166833 to 185899 of strain 309 complete genomes.

Throughout this study we use the following designations: gene number = MPN130, gene name = *mpn130*, and protein name = Mpn130.

## Results

### Two sequence variations of *mpn137*-*mpn138* region are observed in *M. pneumoniae* strains

**Figure 1:**
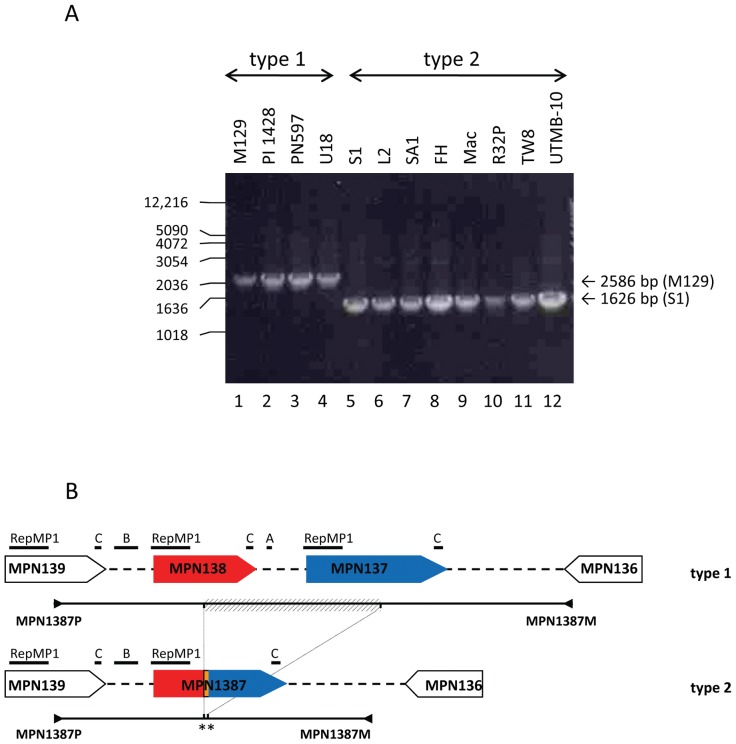
Sequence variations involving MPN137 and MPN138. **A. PCR amplification of MPN138/7 regions.** Chromosomal DNA of four type 1 and eight type 2 strains was used, and generated products were electrophoresed on 1% agarose. Amplicon size was estimated using 1-kb DNA ladder. **B. Comparison of MPN137 and MPN138 regions in different clinical strains.** The analyzed region contains loci MPN139 (open arrow), MPN138 (red arrow) and MPN137 (blue arrow) in M129 strain and all tested type 1 isolates. The fused reading frame (MPN138/7, red and blue arrow) was found in all tested type 2 strains. The location of RepMP1-core elements and short repeats B and C within analyzed regions is indicated. The position of amplified genomic regions (line) and primers (▸,◂) is shown for both type 1 and type 2 strains. The region deleted in type 2 strains is presented (striped bar) and the 49 nt-region originally not found in either MPN137 or MPN138 is indicated (orange stripe, **).

We set out to categorize sequence variations among a set of 30 *M. pneumoniae* strains by amplifying chromosomal regions containing *mpn138* and *mpn137* genes. The assembled set included reference strains B9-M129 (type 1), FH (type 2) [9], other *M. pneumoniae* strains deposited in ATCC as well as clinical strains of different geographic origins collected at different times by us and others (Table 2). PI1428, PN -and U-series generated a ∼2.6 kb PCR product similar to M129 whereas L2, SA1, FH, Mac, R32P, UTMB-10, and strains of TW series yielded ∼1.6 kb amplicons that matched with a PCR product generated for S1 isolate (Figure 1A).

**Table 2:** *M. pneumoniae* isolates used in the study.

Strain	Origin	Yr(s) of isolation	Reference
Type 1			
B9-M129^a^	North Carolina	1968	ATCC29342
PI1428			ATCC29085
U series^b^	Canada	1992–1994	[37]
PN series^c^	Washington	1964, 1969, 1974	[38]
Type 2			
S1	Texas	1993	[5]
L2	Texas	1993	unpublished
SA1	Texas	2001	[39]
Mac	California	1944	ATCC15492
FH	California	1959	ATCC15531
TW series^d^	South Carolina	1974	[40]
UTMB-10	Texas	1986	ATCC49894
R32P	South Carolina	1974	[40]

a M129 obtained from ATCC and maintained in the laboratory (designated B9-M129) was used in amplification reactions.

b Includes strains PN597, PN6644 and PN 14366.

c In 1995 isolation of 24 strains of *M. pneumoniae* from urogenital specimens patients was reported [37]. Fifteen strains were included in this study (U2, U9, U13, U14, U15, U16, U18, U19, U20, U22, U23, U24, U28, U24 and U37).

d Includes strains TW7, TW8 and TW48.

PCR products from all nine S1-like strains and from five randomly chosen M129-like isolates were sequenced. In all, sequencing of S1-like strains (Figure 1B) revealed the matching replacement of 888 nucleotides with a novel 49-nucleotide segment. Since the replaced segment contained *mpn138*-*mpn137* intergenic region, residual portions of *mpn138* and *mpn137* were linked to each other, composing a hybrid gene (*mpn138/7*) of 576 nucleotides as described by us [5].

### *mpn130* is absent in all analyzed S1-like strains

**Figure 2:**
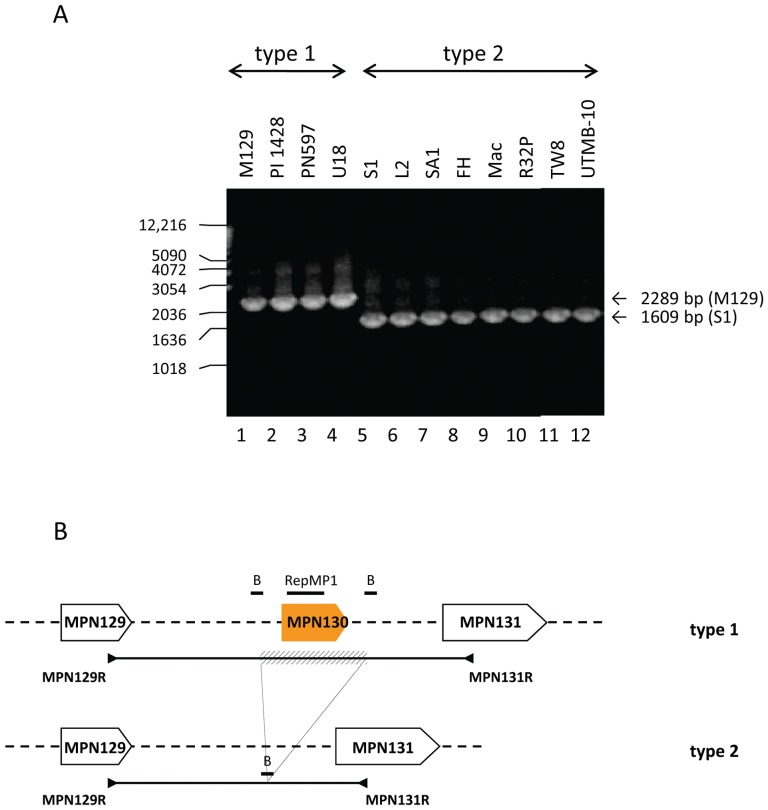
Type-specific deletion of MPN130. **A. PCR amplification of MPN129 to MPN131 regions.** Chromosomal DNA of four type 1 and eight type 2 strains was used and generated products were visualized on 1% agarose for analysis. Amplicon size was estimated using 1-kb DNA ladder. **B. Comparison of MPN129-MPN131 regions among** ***M. pneumoniae*** **clinical strains.** In reference strain M129 and other type 1 isolates, the RepMP1-containing gene (MPN130, orange arrow) is located between coding regions MPN129 and MPN131. In all tested type 2 strains, MPN130 is missing. The location of RepMP1-core element and sRepB within analyzed regions is indicated. The position of amplified genomic regions (line) and primers (▸,◂) is shown for both type 1 and type 2 strains, and the region deleted in type 2 strains is presented (striped bar).

We reported that the 49-nucleotide region identified within *mpn138/7* matched completely a region within *mpn130*, a gene that was absent in clinical strain S1 [5]. Chromosomal regions containing *mpn130* and the adjacent genes (Figure 2) were therefore amplified from all strains and compared to M129 (2289 bp) and S1 (1609 bp) amplicons. All M129-like strains yielded ∼2.3 kb size product and all S1-like strains generated ∼1.6 kb size product (Figure 2A). Subsequent sequencing of the 1.6 kb amplicons revealed the identical 680-bp deletion in all tested S1-like strains (enclosing *mpn130* together with 116 nucleotides of up- and 141 nucleotides of downstream regions; Figure 2B) [5].

### Two novel sReps associated with RepMp1-core are identified

**Figure 3:**
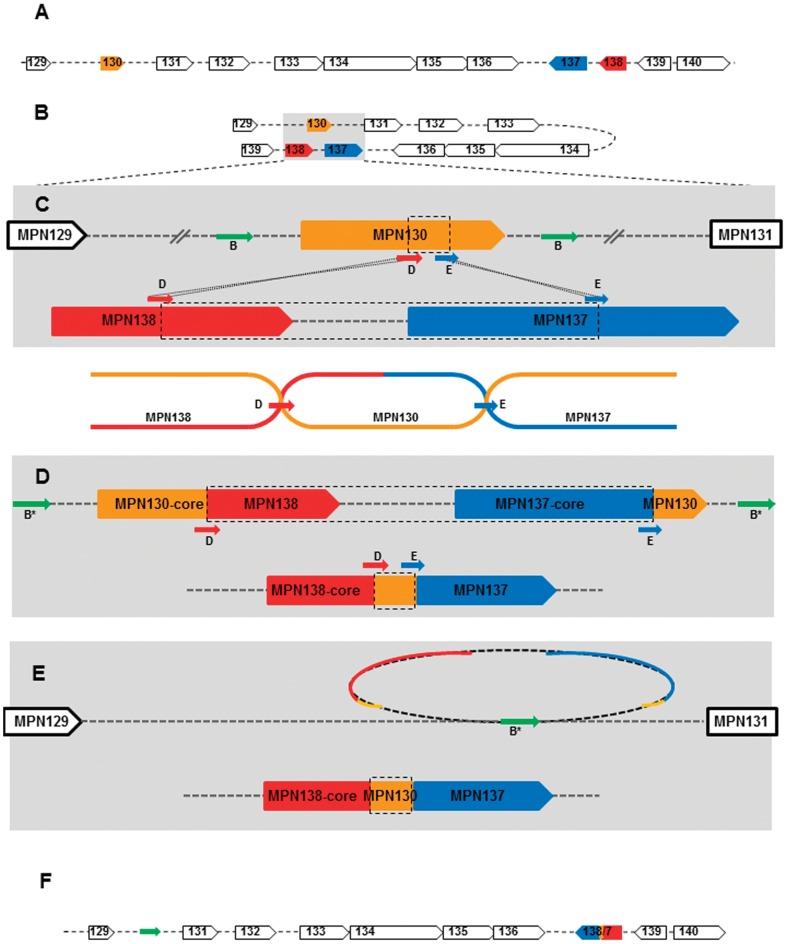
Model of two proposed recombination events. **A and B. Chromosomal region MPN129-MPN140 in** ***M. pneumoniae*** **M129.** Position, length and orientation of all genes are presented and color-coded as above (arrows). **C. Identification of sReps within MPN130, MPN137 and MPN138.** Short repeats B (green arrows labeled B) were identified within both intergenic regions surrounding MPN130. Analyses revealed copies of sRepD and sRepE (red and blue arrows labeled D and E, respectively). Corresponding sReps involved in homologous recombination are connected by dotted lines. A diagram illustrates exchange of chromosomal regions during homologous recombination. **D. Chromosomal regions after homologous recombination.** Coding regions of three RepMP1-genes with rearranged domains are shown. sRepBs presumably involved in sequence deletion are indicated (asterisks). **E. Deletion of two RepMP1-genes.** Deleted region containing two RepMP1-genes (dotted loop) and implicated sRepB (green arrow) are represented. **F. Chromosomal region MPN129-MPN140 in S1.** Detailed depiction of MPN129-MPN131 and MPN138/7 regions and organization of the chromosomal region are presented.

Since we detected major identical sequence rearrangements involving *mpn130*, *mpn137* and *mpn138* genes in all S1-like strains, we analyzed these genes and their immediate surroundings for the presence of recombination favoring short repeats (both direct and inverted, Table 3, Figure 3A and 3B). Analysis of the chromosomal region between genes *mpn129* and *mpn140* in M129 strains revealed copies of short repeats sRepA, sRepB and sRepC that were previously associated with RepMP1-core elements [17]. As indicated in Figures 1B and 2B, due to deletion, S1-like strains lack several of these short repeats. In particular, M129-*mpn130* is flanked by two direct sRepB repeats (72-nt and 69-nt, Table 3, Figure 2B, 3C and 3D). In the corresponding chromosomal region in S1 contains only one sRepB repeat (Figure 2B and 3E; Table 3). Further examination of M129-*mpn130*, *mpn138* and *mpn137* sequences led to the identification of two additional short repeats designated sRepD (46-nt) and sRepE (41-nt). In M129, inverted sRepD is present within *mpn130* and *mpn138* and inverted sRepE is identified within *mpn130* and *mpn137* (Table 3, Figure 3C). Notably, the region of M129-*mpn130* enclosed between sRepD and sRepE is the 49-nucleotide linker of *mpn138* and *mpn137* in S1-*mpn138/7* (Figure 3E).

**Table 3:** Short repeats identified within *mpn129*-*mpn139* region of *M. pneumoniae*.

Repeat		Position^a^	Length (nts)	Location
**A** b		178270 to 178346	77	*mpn137*-*mpn138* intergenic region
**B** b	direct	168863 to 168920	58	upstream of MPN130
		169544 to 169600	57	downstream of MPN130
		179019 to 179073	55	downstream of MPN139
		179723 to 179778	56	upstream of MPN139
**C** b		169359 to 169433	75	within *mpn130*
**D**	invert	169243 to 169288	46	within *mpn130*
		178691 to 178646	46	within *mpn138*
**E**	invert	169324 to 169364	41	within *mpn130*
		177771 to 177731	41	within *mpn137*

a Position within M129 genome.

b BLAST analysis of M129 genome was done using previously published sequence of sRepA, sRepB and sRepC [17].

### Typing of *M. pneumoniae* strains is done by analysis of P1 genes

Analysis of 28 additional strains revealed two conserved sequence variants when chromosomal regions containing *mpn130*, *mpn137* and *mpn138* were compared. This observation prompted us to look at the variation in other RepMP elements. Based on the RepMPs within *mpn141* gene (RepMP2/3, RepMP4), nineteen strains were classified as type 1 and nine as type 2 (Table 2). Interestingly, all S1-like strains (as defined by *mpn130*, *mpn137* and *mpn138* organization) were classified as type 2, whereas all M129-like isolates exhibited restriction patterns characteristic of type 1. Clearly, detected RepMP1-associated sequence differences are type specific. Hereafter the S1-like and M129-like strains will be called type 2 and type 1, respectively, to maintain established nomenclature.

We also amplified and evaluated the *mpn142*-RepMP5 regions from all collected isolates (data not shown). As expected, all type 1 strains yielded ∼2 kb amplicons (1979 bp based on primers position in M129: RepMP5-1) and all type 2 strains yielded ∼200 bp shorter product (RepMP5-2; 1778 bp based on the sequence of this region in FH, S1 and 309 DNA) [12].

### PCR amplification suggests conservation of RepMP1-genes in isolates

To examine the possible involvement of other RepMP1s in sequence variation, we performed BLAST analysis of the complete M129 genome sequence using sequences of all fourteen previously reported RepMP1 core elements. Twenty full-length or partial copies of the repeat were identified throughout the chromosome (Table S2).

Apart from *mpn130*, *mpn137* and *mpn138*, the remaining seventeen RepMP1-genes were amplified from the compiled *M. pneumoniae* strains using specific primers (Table S1), and the generated products were compared with M129-products. All amplifications yielded expected products (data not shown) confirming their presence and conservation.

### Type-specific variations in tandem repeat number are identified within the coiled coil DUF16 domain of *mpn524*, *mpn137* and *mpn127*

Sequence analysis of all *mpn138/7* genes (type 2 strains) revealed that downstream of the fusion (i.e., within the residual *mpn137*), twenty one nucleotides were missing from the stretch of seven 21-nucleotide tandem direct repeats (nucleotides 595 to 615 in M129-*mpn137*; TCGCCTTGATTCTGTTGAAGG).

In all type 1 strains, *mpn524*–containing regions yielded a slightly larger PCR product than all type 2 strains. Nucleotide sequencing revealed the deletion of twenty one nucleotides in all type 2-*mpn524* (AAAAAATGGACAAGATGGAAG; nucleotides 395 to 415 in M129-*mpn524*). Closer evaluation of the M129-*mpn524* sequence showed four 21-nucleotide tandem direct repeats within the open reading frame (nucleotides 359 to 436) whereas all type 2-*mpn524* retained only 3 tandem repeats.

Similarly, amplification of *mpn127* regions yielded products of two different sizes. All type 1 strains contained sequences identical to M129-*mpn127*. Sequencing of the products generated from type 2 isolates showed that their *mpn127* contains an identical 42-nt insertion (TTGGTTT CAATGGAAAGCCGTCTTGATTCTATGGAAAATCGC) localized 618 nucleotides downstream of the predicted *mpn127*-start codon. Unexpectedly, the insertion was detected downstream of the predicted MPN127 (M129-MPN127 is 543 nucleotides long, nucleotides 164484 to 165026, and encodes 180 amino acids). We sequenced the PCR product generated from B9-M129 strain and showed that the coding region of *mpn127* was 774 nucleotides long and contained 8.5 tandem direct 21-nt repeats (region 567 to 744) (Figure. S3). On the other hand, coding region of the type 2-*mpn127* with 42-nt insertion was 816 nucleotides long with two additional 21-nt repeats (Figure S4 and S5).

### Variation in repeat number within *mpn501* is not strain type-specific

Two different size PCR products were generated when *mpn501* region was amplified. All type 1 strains and three type 2 isolates (S1, L2 and Mac) yielded amplicons that were the same size of M129 (767 bp), while PCR products generated from the remaining type 2 strains (i.e., FH, R32P, SA1, TW7, TW8, TW48, and UTMB) appeared larger. Further sequencing analysis revealed the presence of 3 perfect 21 nucleotide tandem direct repeats (ATGGAAGTAAAAATGGACAAA) in M129-*mpn501* that start at position 493, whereas all larger amplicons contained an additional full 21-nucleotide repeat within this region that resulted in a PCR product of 788 bp long.

### Number of direct tandem repeats for all members of DUF16 protein family was tested

Since the variation in the 21 nucleotide tandem direct repeats were observed within the coiled coil region (DUF16) of RepMP1, six more genes were included in the analysis of repeat numbers. These six genes *mpn010*, *mpn013*, *mpn038*, *mpn104*, *mpn145*, and *mpn675* did not contain the RepMP1-core element but encoded proteins with distal coiled-coil domains (DUF16) (Table S2). No variation in size of generated products was observed among the strains.

## Discussion

The *M. pneumoniae* genome contains four types of large repetitive elements (RepMP1, RepMP2/3, RepMP4 and RepMP5; Table 1) which constitute 8% of the M129 genome. RepMP2/3, RepMP4 and RepMP5 have been studied extensively as they are found within genes that encode major virulence factors of *M. pneumoniae* (*mpn141* and *mpn142*, adhesin P1 and cytadherence-related proteins, respectively). Sequence divergence within RepMP2/3 and RepMP4 allows the classification of all worldwide strains into two groups. Similar repeats were also detected within adherence related genes of *M. genitalium* (*mg191* and *mg192*) and *M. gallisepticum* [14], [15], [16]. It has been assumed, and demonstrated in case of *M. genitalium* [14], [15], that homologous recombination among numerous copies of these repeats allows for sequence variations among strains.

In this study we focused on the *M. pneumoniae*-specific RepMP1 sequence element and its role in generating sequence divergence among clinical isolates. RepMP1-proteins belong to the largest *M. pneumoniae* protein family that is united by the coiled-coil domain (DUF16) within their distal regions (Table 1 and S2) [25]. RepMP1-proteins form a subset of this protein family that also shares different degrees of homology within their proximal regions. Sequence similarities of the proximal domains result from the RepMP1-core element that, in most cases, is localized within the 5′-end of the gene (Figure S1 and S2). The sequence of the distal domains (DUF16) is not conserved, and domains differ in amino acid residues and length. The important common feature of DUF16 domains is the presence of direct tandem 7-aa repeats that mediate its coiled-coil structure [25].

Through analysis of 31 *M. pneumoniae* isolates, including genome sequence of *M. pneumoniae* strain 309 we clearly demonstrate a major recombination event associated with three RepMP1-genes (*mpn130*, *mpn137* and *mpn138*). Recombination produced a hybrid gene (*mpn138/7*) that has the proximal region (or RepMP1-core) of *mpn138* and the distal region (or DUF16 domain) of *mpn137* joined together through a 49-nt remnant of a third gene (*mpn130*).

Since we detected identical sequence rearrangements involving *mpn130*, *mpn137* and *mpn138* genes in all type 2 strains, we investigated *mpn129*-*mpn140* chromosomal regions for short repeats associated with RepMP1-core. Completed analysis revealed several copies of all five sReps (Table 3, Figure 1B, 2B). Based on identified sequence differences and on the position of the short repeats sRepB, sRepD and sRepE, we propose the occurrence of two subsequent events (Figure 3). First, homologous recombination lead to the exchange of chromosomal regions enclosed between sRepDs and sRepEs (Figure 3C, 3D). As a result, RepMP1-cores and DUF16 domains were rearranged in these three genes. In the second step, the recombination between the direct repeats sRepB resulted in deletion of the region enclosed between them (Figure 3E). Thus, in place of three genes (*mpn130*, *mpn137* and *mpn138* as observed in M129) only one gene was retained (*mpn138/7* as described for all type 2 strains).

The presence of numerous RepMP1-core elements within *M. pneumoniae* genomes prompted us to look for and evaluate the short repeats (sReps) within the genome, as they seem to be involved in intergenic recombination of domains and deletion mechanisms. BLAST analysis of M129 genome revealed numerous copies of all five sReps (A–E) and their association with RepMP1- and DUF16-genes (encoded proteins contain the DUF16 domain but not RepMP1-core) (Table S2). Short repeats A and B were found exclusively within intergenic regions adjacent to these genes. All three remaining sReps are localized within coding regions. While copies of sRepD are found within the conserved domain of several genes (3′-end of the RepMP1-core element), sRepC and sRepE are found within the coiled-coil region of several RepMP1-genes (i.e., *mnp094*, *mnp100*, *mnp204*, *mnp501*, etc.).

**Figure 4:**
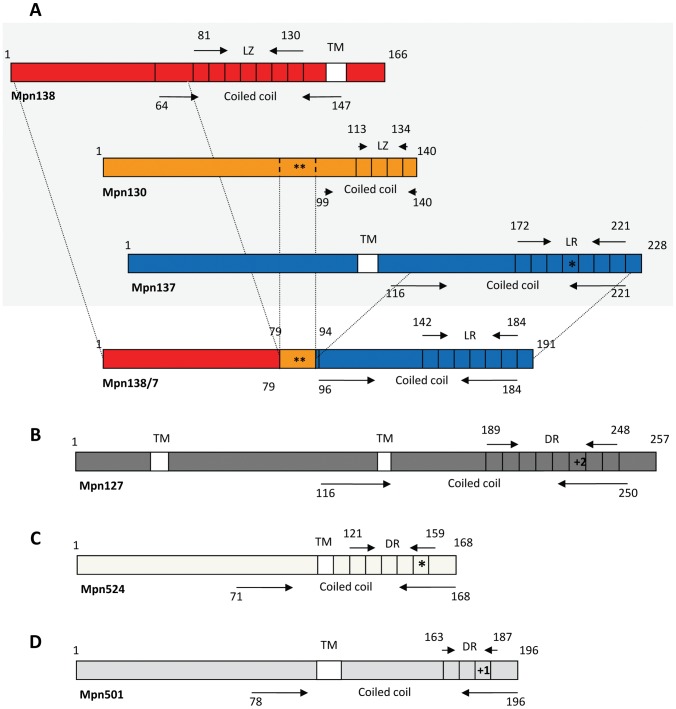
Predicted secondary structure of modified RepMP1-proteins. **A. Type-specific modification of Mpn130, Mpn137 and Mpn138 proteins.** Three proteins (within grey box) are predicted in type 1 isolates. In type 2 strains, fused protein Mpn138/7 consists of Mpn138-N-terminal and Mpn137-C-terminal region that is shorter by one heptad repeat (*). Only the indicated 16-aa region of MPN130 (**) is retained in type 2 strains. Regions of coiled coils are shown (numbers represent start and end of region). Locations of leucine zipper (LZ) in Mpn138 and Mpn130 and of leucine repeats (LR) in Mpn138 and Mpn138/7 are indicated. **B. Type-specific modification of Mpn524.** The position of direct tandem heptad repeats (DR) and coiled coil region is presented for type 1 Mpn524. In all type 2 strains one heptad repeat is deleted (*). **C. Mpn501 modification.** The positions of direct tandem heptad repeats (DR) and coiled coil domains are indicated. The insertion of an additional heptad repeat (+1) is the only not type-specific modification identified among strains. Transmembrane domains (TM) were predicted by DAS analysis.

Analyses and cross-comparison of RepMP1-genes/proteins lead us to the conclusion that RepMP1-core elements and sReps provide a network for intergenic domain exchanges. For example, as demonstrated in Figure 3D, sRepD and sRepE-mediated recombination among three genes leads to three novel genes/proteins with different combinations of conserved proximal regions with coiled-coil domains (Figure 3 and 4). It is predictable that the exchange of domains will provide proteins with modified function. Currently, function(s) of both conserved and DUF16 domains as well as the majority of RepMP1-proteins remain unknown. So far, it has been shown that transposon insertions within MPN104 and MPN524 resulted in *M. pneumoniae* mutants with altered satellite growth phenotype and altered gliding motility, possibly suggesting these proteins could play a role in cytoskeletal functions [26]. Recombination-mediated protein domain variations have been reported previously for the Arp protein (an immunoglobulin A receptor in the M protein family) of *Streptococcus pyogenes* [27]. Repeat-associated plasticity in the *Helicobacter pylori* RD gene family has been analyzed, and a mechanism leading to the exchange of domains was proposed [28]. In eukaryotes, these translocations often involve transcriptional factors [29], [30].

Variability in the number of tandem repeats within the DUF16 domain is commonly observed in several RepMP1-proteins (Figure 4, Table 4). Such modifications in repeat numbers could likely result from slipped-strand mispairing events combined with unequal crossovers. In contrast to type 1 strain M129, one of the 21-nt tandem direct repeats is deleted in all type 2- *mpn138/7* and *mpn524*. Due to this deletion all Mpn138/7 fused proteins are missing a 7-aa tandem repeat when compared with M129-Mpn137 putative protein sequence (V^160^EGRLDS, Figure 4, Table 4). Likewise, the type 2-specific Mpn524 protein is missing seven amino acids (E^132^KMDKME, Table 4). Furthermore, the type 2-specific Mpn127 protein contains an additional fourteen residues when compared with M129-Mpn127 (amino acids RLVSMESRLDSMEN inserted after N^206^, Figure 4, Table 4). Similarly, three type 2 strains possess a Mpn501 protein with an additional 7-aa repeat (residues VKMDKME inserted after E^187^, Figure 4, Table 4). All these changes are found within coiled-coil regions of the proteins and likely impact on their structure and function (Figure 4). For instance, the coiled coil region of the fused Mpn138/7 protein is not recognized as a Leucine zipper (found in the M129-Mpn138) (Figure 4). Insertion of additional seven residues within coiled-coil region of Mpn501 might lead to the loss of the transmembrane domain (TM) (Figure 4).

**Table 4:** Summary of RepMP1-gene sequence variability in analyzed *M. pneumoniae* strains.

Gene	Nucleotides (amino acids) changes as compared with M129 genome
	**A. Type 2-specific sequence variations**
**MPN127**	42 nucleotides are inserted after MPN129-MPN127-T^629^. As a result, encoded type 2-proteins contain two additional heptad tandem repeats within their coiled-coil domain.
**MPN130**	In type 2 strains, 680-bp deletion containing entire MPN130 reading frame was found. Only 49-nucleotide region of MPN130 is retained in the fused gene MPN138/7.
**MPN137**	Nucleotides 373 to 687 remain as portion of MPN138/7 coding region. Additionally, twenty one nucleotides are missing from the retained sequence (nucleotides 595 to 615 of M129-*mpn137*). The maintained sequence encodes coiled-coil domain of fused protein in which one of the direct heptad tandem repeats is missing.
**MPN138**	Nucleotides 1 to 233 are fused with the remaining portion of MPN137. The resulting fused coding region (576 bp) encodes a 191-aa protein, Mpn138/7, present only in type 2 strains.
**MPN524**	One of 21-nucleotide tandem repeats (nucleotides 395 to 415 of M129-*mpn524*) are missing. As a result, a 7-aa tandem repeat is missing from the coiled-coil region of encoded protein.
	**B. Sequence variations not related to the isolate type**
**MPN501**	In several strains regardless of their type, an additional 21-nucleotide repeat is inserted after A^561^. As a result, the encoded protein contains an additional heptad tandem repeat within the coiled-coil domain.

Recently, the numbers of tandem repeats within MPN501 and MPN524 were evaluated as part of a multi-locus variable-number tandem-repeat analysis (MLVA) of nearly 340 *M. pneumoniae* strains originating from Tunisia, Japan, Germany, England, Wales, and other European countries [31], [32], [33]. In the analyzed strains, the number of MPN501-repeats varied from four to six while the number of MPN524-repeats fluctuated from six to eight and variations were not type specific. Data were presented that tandem repeat numbers did not change during strain passage in broth culture and, possibly, in the course of persistent infection. Our analysis of MPN501 and MPN524 revealed comparable numbers of tandem repeats. We observed type-specific differences in the numbers of tandem repeats within MPN524, as well as within MPN1387 and MPN127.

In conclusion, numerous copies of RepMP1-core elements and associated short repeats are spread throughout the *M. pneumoniae* genome, creating a network for gene rearrangement through homologous recombination. Still, we identified only a singular identical recombination of the same three RepMP1-genes in all type 2 isolates. Impressively, regardless of the presence of this intricate network, our data provide further evidence for the existence of two highly conserved groups of *M. pneumoniae* strains as demonstrated in the past [12], [34], [35]. Previous experiments clearly indicate that type-specific combinations of the repetitive elements in the P1 and *mpn142* genes are not essential for the successful adherence of *M. pneumoniae* to host cells and the colonization of the respiratory tract of guinea pigs [36]. Therefore, *M. pneumoniae* virulence does not seem to be considerably influenced by the strictly defined combination of repetitive elements and further studies are required to explain and understand reason(s) behind this lack of sequence divergence.

## Supporting Information

Figure S1 **RepMP1-genes and corresponding core elements.** Twenty RepMP1-genes of M129 strains (black arrows) and their core elements (grey arrows) are presented. The length and orientation of the arrows reflect actual sequences and their positions. Different panels group genes based on the homologies of corresponding core elements (A to D; colors represent different levels of homologies among these four groups). Panel E contains two genes with core element sequences in opposite to the coding region orientation.
(TIF)

Figure S2 **Alignment of RepMP1-proteins.** Proteins were aligned using CLUSTAL X. Groups of proteins with homologous conserved domains are highlighted. Proteins Mpn037 and Mpn465 were not included in alignment.
(TIF)

Figure S3 **Sequence of MPN127 region in** ***M. pneumoniae*** **strain B9-M129 maintained in the laboratory.**
(DOCX)

Figure S4 **Sequence of MPN127 region in clinical** ***M. pneumoniae*** **strain S1.**
(DOCX)

Figure S5 **Sequence of MPN127 region in FH** ***M. pneumoniae*** **strain maintained in the laboratory**
(DOCX)

Table S1 **Primers used for PCR amplification of MPN142-RepMP5 repeat and RepMP1-genes.**
(DOCX)

Table S2 **RepMP1 and DUF16-containing genes and their position in** ***M. pneumoniae*** **M129 genome.**
(DOCX)
