# Supplementary figures and images for: Mycoplasma pneumoniae Large DNA Repetitive Elements RepMP1 Show Type Specific Organization among Strains
Source: PLoS One. 2012 Oct 16;7(10):e47625. doi: 10.1371/journal.pone.0047625 (PMC3472980; doi:10.1371/journal.pone.0047625)

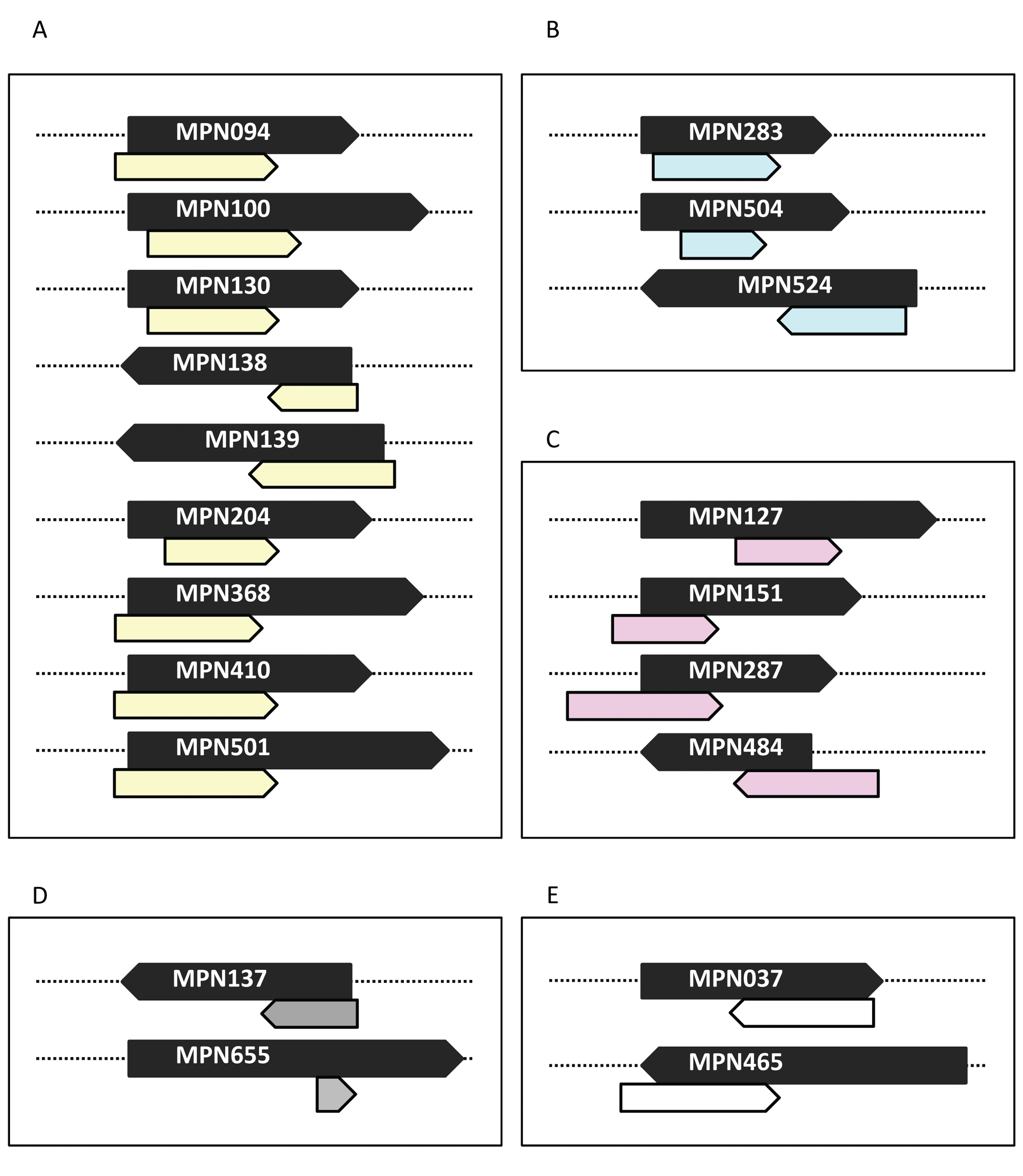

Supplement: Figure S1 — RepMP1-genes and corresponding core elements. Twenty RepMP1-genes of M129 strains (black arrows) and their core elements (grey arrows) are presented. The length and orientation of the arrows reflect actual sequences and their positions. Different panels group genes based on the homologies of corresponding core elements (A to D; colors represent different levels of homologies among these four groups). Panel E contains two genes with core element sequences in opposite to the coding region orientation. (TIF) [file pone.0047625.s001.tif]

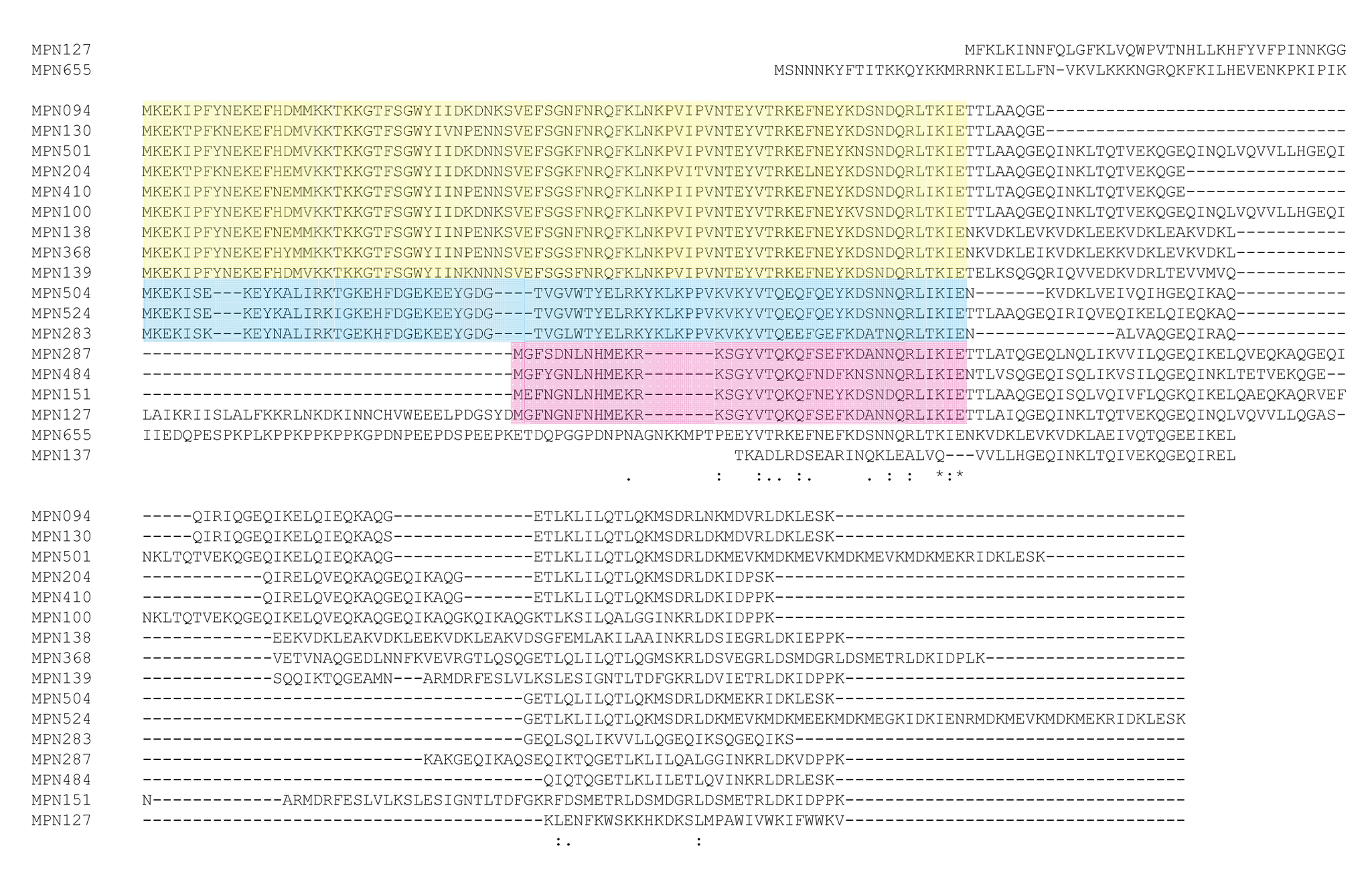

Supplement: Figure S2 — Alignment of RepMP1-proteins. Proteins were aligned using CLUSTAL X. Groups of proteins with homologous conserved domains are highlighted. Proteins Mpn037 and Mpn465 were not included in alignment. (TIF) [file pone.0047625.s002.tif]
