# Supplementary material for: Mycoplasma pneumoniae Large DNA Repetitive Elements RepMP1 Show Type Specific Organization among Strains
Source: PLoS One. 2012 Oct 16;7(10):e47625. doi: 10.1371/journal.pone.0047625 (PMC3472980; doi:10.1371/journal.pone.0047625)
Supplement: Figure S3 — Sequence of MPN127 region in M. pneumoniae strain B9-M129 maintained in the laboratory. (DOCX) [file pone.0047625.s003.docx]

B9-M129-MPN127

Sequence of MPN127 region in *M. pneumoniae* strain B9-M129 maintained in the laboratory

Length: 820 nts

Coding region: 774 nts (47 to 820)

Protein: 257 aa

>B9-M129-MPN127 GTGTCTTTACTAACTTAAAGCAATTTAGTTAGTGTAAAGACTCATAATGTTTAAATTAAAAATTAACAACTTCCAATTAGGTTTCAAACTCGTTCAGTGACCAGTGACGAACCACCTTCTTAAACATTTTTACGTTTTTCCAATCAATAATAAAGGAGGTCTTGCCATTAAACGCATAATTTCACTTGCTTTGTTTAAGAAAAGACTTAACAAAGATAAGATTAATAATTGTCATGTTTGGGAAGAAGAGTTACCTGATGGTAGCTACGACATGGGATTTAATGGCAATTTCAACCATATGGAAAAACGAAAAAGTGGTTATGTTACCCAAAAGCAGTTTAGCGAGTTCAAAGATGCCAACAATCAGCGTCTCATAAAGATTGAAACTACTTTGGCTATCCAAGGCGAACAAATCAACAAATTGACTCAAACTGTTGAAAAGCAAGGCGAACAAATCAATCAATTAGTTCAAGTTGTGCTTCTTCAGGGCGAGCAAATTAGAGAACTTCAAGTGGAGCAAAAAGCACAAAGACAAGAGTTTAATGCCCGCATGGATCGTTTGGAAAATCTTTTGGTGGAAAGTATAGAATCTACCAATAAGCGCTTCGACTCTATGGAAAGACGTTTAGACTCTATGGATAGTCGTCTTGATTCTATGGAAAATCGCTTAGATTCAATGGAAGGTCGTCTTGATTCTATGGAAAATCGCTTAGATTCAATGGAAGGTCGTCTTGATTCTGTTGAAGGACGCTTAGACTCTATGGAAACTCGTTTAGACTCTATGGAAACTCGCCTGGACAAAGTCGATCCGCCCAAATAG

MFKLKINNFQLGFKLVQWPVTNHLLKHFYVFPINNKGGLAIKRIISLALFKKRLNKDKINNCHVWEEELPDGSYDMGFNGNFNHMEKRKSGYVTQKQFSEFKDANNQRLIKIETTLAIQGEQINKLTQTVEKQGEQINQLVQVVLLQGEQIRELQVEQKAQRQEFNARMDRLENLLVESIESTNKRFDSMERRLDSMDSRLDSMENRLDSMEGRLDSMENRLDSMEGRLDSVEGRLDSMETRLDSMETRLDKVDPPK
