# Supplementary material for: Mycoplasma pneumoniae Large DNA Repetitive Elements RepMP1 Show Type Specific Organization among Strains
Source: PLoS One. 2012 Oct 16;7(10):e47625. doi: 10.1371/journal.pone.0047625 (PMC3472980; doi:10.1371/journal.pone.0047625)
Supplement: Figure S4 — Sequence of MPN127 region in clinical M. pneumoniae strain S1. (DOCX) [file pone.0047625.s004.docx]

S1-MPN127

Sequence of MPN127 region in clinical *M. pneumoniae* strain S1.

This sequence overlaps (underlined nucleotides) with the sequence previously submitted for this strain (EF470909).

1. Length: 3140 nucleotides
2. partial MPN126: 1 to 211 (211 nucleotides)

69 aa

LVDQAKSYLCDVLIVGHSHIEHYETIDGIQVINPGSLEIPRNPRKLPTYCNLNLSQGRISDLTFHFPRD

1. MPN127: 602 to 1417 (816 nucleotides)

271 aa

MFKLKINNFQLGFKLVQWPVTNHLLKHFYVFPINNKGGLAIKRIISLALFKKRLNKDKINNCHVWEEELPDGSYDMGFNGNFNHMEKRKSGYVTQKQFSEFKDANNQRLIKIETTLAIQGEQINKLTQTVEKQGEQINQLVQVVLLQGEQIRELQVEQKAQRQEFNARMDRLENLLVESIESTNNRFDSMERRLDSMDSRLDSMENRLVSMESRLDSMENRLDSMEGRLDSMENRLDSMEGCLDFVEGRLDSMETRLDSMETRLDKVDPPK

1. MPN128: 2961 to 3140 (450 nucleotides)

149 aa

MFGLKVKNAEADTAKSNEKLQGAEATGSSTTSGSGQSTQRGGSSGDTKVKALQVAVKKKSGSQGNSGDQGTEQVELESNDLANAPIKRGSNPASPTQGSRLRHHPIQFGIWSIRHPHPLKAVACDRANSQGPPQMIRLDPHSVRCALCL

>S1-MPN126-MPN128

GCTGGTTGATCAAGCTAAAAGCTATCTTTGTGATGTTTTAATTGTGGGTCACAGTCACATAGAGCACTATGAAACTATTGATGGGATCCAGGTTATTAACCCCGGTTCTCTAGAAATTCCCCGTAATCCGAGAAAGCTACCTACCTACTGTAATTTAAACTTAAGTCAGGGCAGGATAAGTGATTTAACGTTCCATTTTCCTCGGGATTAAGTCAAATAAATAAAAAAAGCCATCTACCTAACAAGCAGAAGGCTAAAAAATACTTTTTTAGCGATATTGAAATTAACAGAAAGATCTGTTAGCAAATGTAAGGAACAAGCTTATGCAAACTTTCAATATGTGACGTGTTGAAAAACCTTTAAGAGTATGCATCAATTGGGAGTAGGAAAGCAGGCTTTCAACACAAGTTTTCGTTTTCCTGCTCCCACTAAATAAATTGGGAAAGGTTTTCAAAGTCGCCAAAAAGATTAAATATTTTTGGAATTTTGAATTTTTAGTTGCTTCTAATTGTTAATAGAGATTTACTTTCAAAAAAACTTTTTGGTTTATTTTTGGTGTCTTTACTAACTTAAAGCAATTTAGTTAGTGTAAAGACTCATAATGTTTAAATTAAAAATTAACAACTTCCAATTAGGTTTCAAACTCGTTCAGTGACCAGTGACGAACCACCTTCTTAAACATTTTTACGTTTTTCCAATCAATAATAAAGGAGGTCTTGCCATTAAACGCATAATTTCACTTGCTTTGTTTAAGAAAAGACTTAACAAAGATAAGATTAATAATTGTCATGTTTGGGAAGAAGAGTTACCTGATGGTAGCTACGACATGGGATTTAATGGCAATTTCAACCATATGGAAAAACGAAAAAGTGGTTATGTTACCCAAAAGCAGTTTAGCGAGTTCAAAGATGCCAACAATCAGCGTCTCATAAAGATTGAAACTACTTTGGCTATCCAAGGCGAACAAATCAACAAATTGACTCAAACTGTTGAAAAGCAAGGCGAACAAATCAATCAATTAGTTCAAGTTGTGCTTCTTCAGGGCGAGCAAATTAGAGAACTTCAAGTGGAGCAAAAAGCACAAAGACAAGAGTTTAATGCCCGCATGGATCGTTTGGAAAATCTTTTGGTGGAAAGTATAGAATCTACCAATAATCGCTTCGACTCTATGGAAAGACGTTTAGACTCTATGGATAGTCGTCTTGATTCTATGGAAAATCGCTTGGTTTCAATGGAAAGCCGTCTTGATTCTATGGAAAATCGCTTAGATTCAATGGAAGGTCGTCTTGATTCTATGGAAAATCGCTTAGATTCAATGGAAGGTTGTCTTGATTTTGTTGAAGGACGCTTAGACTCTATGGAAACTCGTTTAGACTCTATGGAAACTCGCCTGGACAAAGTCGATCCGCCCAAATAGTTTTTAGTTAAAAAATAAGGGTATCAGCAGTTAATCTTAAAGATTAGTTGCTGGTATTTTTATTCATTTAATGCTTTCTAAAAACTTTTTGTCAATTTTGTTGGCACTTCTAAATTGCTAGTTCAATTTATTAAGTGGAGGAGCAAAATGCAAATCTTTAATTTTTGAAATTGATCAGTAAAAAATCTGATCACCGTTGGTAGCATTGCTGCAAATAGTAGTTATGTAATTTGCAAACAGAAAGGAATTTTTCAATATGAGTAATACAAAAAAATTAAGAATAACTTCAATCGAAGAAACAATAGAAATAATAGTTAATTCAAAACTATTAGCTTTTGGTTTTTACCCAGTTGGTTTAGAAGATGGAACTTACGAACTTAATGTAAGGATTATTTTTAAAGAAAAAACTGCAGATTCTGAGAAGAGTAGCAGTGTACATCCTTTACAAAAGGAGTATTATGTAAGGGAATTTTTGAGATTTCTTGAGCGCCATAAAGATTCTCTGGCGCAACAACTATTAGTAGTAGAAGACAATTAAATGATTTCTCCCAAACTGGATTGACTCATCCTCACCGCCACCGCCTCCCTCGCGGCGGGACTCACCGTAGTGGGACACTTCACAAGTACCACCACGACGCTCAAGCGCCAGCAATTTAGCTACACCCGCCCTGACGAGGTCGCGCTGCGCCACACCAATGCCATCAACCCGCGCTTAACCCCGTGAACGTATCGTAACACGAGCTTTTCCTCCCTCCCCCTCACGGGTGAAAACCCCGGGGCGTGGGCCTTAGTGCGCGACAACAGCGCTAAGGGCATCACTGCCGGCAGTGGCAGTCAACAAACCACGTATGAACCTGCGCGAACCGAAGCGGCTTTGACCGCATCAACCACCTTTGCGTTACGCCGGTATGACCTCGCCGGGCGCGCCTTATACGACCTCGACTTTTCGAAGTTAAACCCACAAACGCCCACGCGCGACCAAACCGGGCAGATCACCTTTAACCCCTTTGGCGGCTTTGGTTTGAGTGGGGCTGCACCCCAACAGTGAAACGAGGTCAAAAACAAGGTCCCCGTCGAGGTGGCGCAAGACCCCTCCAATCCCTACCGGTTTGCCGTTTTACTCGTGCCGCGCAGCGTGGTGTACTATGAGCAGTTGCAAAGGGAGTTGGGCTTACCACAGCAGCGAACCGAGAATGGTCAAAATACTTCCACCACCGGGGCATTACCACAGCAGCGAACCGAGAATGGTCAAAATACTTCCACCACCGGGGCAATGTTTGGCTTGAAGGTGAAGAACGCCGAGGCGGACACCGCGAAGAGCAATGAAAAACTCCAGGGCGCTGAGGCCACTGGTTCTTCAACCACATCTGGATCTGGCCAATCCACCCAACGTGGGGGTTCGTCAGGGGACACCAAAGTCAAGGCGTTGCAGGTGGCGGTGAAAAAGAAATCCGGGAGTCAGGGCAACTCCGGTGACCAAGGCACCGAACAGGTGGAACTTGAATCTAATGATTTAGCCAACGCCCCGATTAAACGGGGCTCCAATCCAGCAAGTCCAACTCAAGGCAGCCGACTTCGGCACCACCCCATCCAGTTCGGAATCTGGTCAATCAGGCACCCCCACCCCCTGAAGGCCGTGGCTTGCGACCGAGCAAATTCACAAGGACCTCCCCAAATGATCCGCCTCGATCCTCATTCTGTACGATGCGCCTTATGCCTTTAA
