# Supplementary material for: Mycoplasma pneumoniae Large DNA Repetitive Elements RepMP1 Show Type Specific Organization among Strains
Source: PLoS One. 2012 Oct 16;7(10):e47625. doi: 10.1371/journal.pone.0047625 (PMC3472980; doi:10.1371/journal.pone.0047625)
Supplement: Figure S5 — Sequence of MPN127 region in FH M. pneumoniae strain maintained in the laboratory (DOCX) [file pone.0047625.s005.docx]

FH-MPN127

Sequence of MPN127 region in FH *M. pneumoniae* strain maintained in the laboratory

Length: 861 nts

Coding region: 816 nts (46 to 861)

Protein: 271 aa

>FH-MPN127

tgtctttactaacttaaagcaatttagttagtgtaaagactcataATGTTTAAATTAAAAATTAACAACTTCCAATTAGGTTTCAAACTCGTTCAGTGACCAGTGACGAACCACCTTCTTAAACATTTTTACGTTTTTCCAATCAATAATAAAGGAGGTCTTGCCATTAAACGCATAATTTCACTTGCTTTGTTTAAGAAAAGACTTAACAAAGATAAGATTAATAATTGTCATGTTTGGGAAGAAGAGTTACCTGATGGTAGCTACGACATGGGATTTAATGGCAATTTCAACCATATGGAAAAACGAAAAAGTGGTTATGTTACCCAAAAGCAGTTTAGCGAGTTCAAAGATGCCAACAATCAGCGTCTCATAAAGATTGAAACTACTTTGGCTATCCAAGGCGAACAAATCAACAAATTGACTCAAACTGTTGAAAAGCAAGGCGAACAAATCAATCAATTAGTTCAAGTTGTGCTTCTTCAGGGCGAGCAAATTAGAGAACTTCAAGTGGAGCAAAAAGCACAAAGACAAGAGTTTAATGCCCGCATGGATCGTTTGGAAAATCTTTTGGTGGAAAGTATAGAATCTACCAATAATCGCTTCGACTCTATGGAAAGACGTTTAGACTCTATGGATAGTCGTCTTGATTCTATGGAAAATCGCTTGGTTTCAATGGAAAGCCGTCTTGATTCTATGGAAAATCGCTTAGATTCAATGGAAGGTCGTCTTGATTCTATGGAAAATCGCTTAGATTCAATGGAAGGTTGTCTTGATTTTGTTGAAGGACGCTTAGACTCTATGGAAACTCGTTTAGACTCTATGGAAACTCGCCTGGACAAAGTCGATCCGCCCAAATAG

MFKLKINNFQLGFKLVQWPVTNHLLKHFYVFPINNKGGLAIKRIISLALFKKRLNKDKINNCHVWEEELPDGSYDMGFNGNFNHMEKRKSGYVTQKQFSEFKDANNQRLIKIETTLAIQGEQINKLTQTVEKQGEQINQLVQVVLLQGEQIRELQVEQKAQRQEFNARMDRLENLLVESIESTNNRFDSMERRLDSMDSRLDSMENRLVSMESRLDSMENRLDSMEGRLDSMENRLDSMEGCLDFVEGRLDSMETRLDSMETRLDKVDPPK
