# Supplementary material for: Mycoplasma pneumoniae Large DNA Repetitive Elements RepMP1 Show Type Specific Organization among Strains
Source: PLoS One. 2012 Oct 16;7(10):e47625. doi: 10.1371/journal.pone.0047625 (PMC3472980; doi:10.1371/journal.pone.0047625)
Supplement: Table S1 — Primers used for PCR amplification of MPN142-RepMP5 repeat and RepMP1-genes. (DOCX) [file pone.0047625.s006.docx]

Table S1: Primers used for PCR amplification of MPN142-RepMP5 repeat and RepMP1-genes.

| \| **Primer** \| **Sequence** \| **Position within M129 genome** \| **PCR product (bp)** \| \| --- \| --- \| --- \| --- \| \| RepMP5F \| CCGCACAGGTATCAGTCAAG \| 185606-185625 \| 1979 (type 1) \| \| RepMP5R \| TACCCCCCATCACTGTCCAA \| 187565-187584 \| 1778 (type 2) \| \| MPN037F \| CCTAATCAATATAGGCACTGTTGG \| 45691-45714 \| 569 \| \| MPN037R \| GCAGTTGATCCTCGTTGACA \| 46240-46259 \|  \| \| MPN094Fa \| GCTGAACTTAGTTGGCAGCA \| 116215-116234 \| 526 \| \| MPN094R \| AATTGCTGGTATTCTTTATTTTGAA \| 116716-116740 \|  \| \| MPN100F \| GCTCAAGCTAAGCTCCCAAA \| 129396-129415 \| 714 \| \| MPN100R \| CGGTGGATGGCTTTTTATTT \| 130090-130109 \|  \| \| MPN127F \| TTTTTGGTTTATTTTTGGTGTCTTT \| 164421-164445 \| 837 (type 1) \| \| MPN127R \| ATTTGGGCGGATCGACTTTGTCCAG \| 165233-165257 \| 879 (type 2) \| \| MPN129F \| TGATGATGTTAGGATCATTAATTGCATTG \| 167990-168018 \| 2289 (type 1) \| \| MPN131R \| GCTGATTAGGCGTAGTTCGCGAACCA \| 170253-170278 \| 1609 (type 2) \| \| MPN138F \| GATTGAAACTGAGTTAAAGAGTCAGGG \| 179355-179381 \| 2586 (type 1) \| \| MPN137R \| ATGAAAGCCGTGGGATCACG \| 176838-176857 \| 1626 (type 2) \| \| MPN139F \| GGTGTACTTGGCTATTCATTGGTG \| 179866-179889 \| 1262 \| \| MPN139R \| CCAGCTTATCAACCTTATTTTCAATC \| 178628-178653 \|  \| \| MPN151Fb \| CGCACAATTTCACTTACTTTGTTTAAG \| 199040-199066 \| 595 \| \| MPN151R \| CTTTTAAAACCAATTTTGGTCGT \| 199612-199634 \|  \| \| MPN204F \| GCAAGTTTTGTTGACACCTTTA \| 247490-247511 \| 764 \| \| MPN204R \| CAAAATCATTAAAAACTAT \| 248225-248243 \|  \| \| MPN283F \| AATGGTATTGATCCCCGTTG \| 336398-336417 \| 483 \| \| MPN283R \| AATTTTCTCGCCCTGCTTTT \| 336861-336880 \|  \| \| MPN287Fb \| CGCACAATTTCACTTACTTTGTTTAAG \| 343665-343691 \| 556 \| \| MPN287R \| CCTTGAGCAGATGGCTTTTT \| 344201-344220 \|  \| \| MPN368Fa \| GCTGAACTTAGTTGGCAGCA \| 439148-439167 \| 715 \| \| MPN368R \| TCCTTGATAGGATGGCTTTTT \| 439842-439862 \|  \| \| MPN410F \| ATACGCCTTTGCGGTGTTAC \| 494604-494623 \| 597 \| \| MPN410R \| AAAAATACCAACAGCTTGTTTTTAAG \| 495175-495200 \|  \| \| MPN465F \| TTAGCCGTCCATCTTTCACC \| 569254-569273 \| 724 \| \| MPN465Ra \| GCTGAACTTAGTTGGCAGCA \| 568550-568569 \|  \| \| MPN484F \| CATCAAAAGCCTGAATCGAA \| 588638-588657 \| 423 \| \| MPN484R \| GATAAATAAAAATACCAACAACTGACA \| 588235-588261 \|  \| \| MPN501F \| CGTTGCTAATGCAAGCTCAA \| 608091-608110 \| 767 or 788 \| \| MPN501R \| CCTTGGACAGATGGCTTTTT \| 608838-608857 \| not type-specific \| \| MPN504F \| AATTTAGCAAAACAGCTGAAC \| 612654-612674 \|  \| \| MPN504R \| AAGGCAAAGATGATGTTAGTCAAA \| 613286-613309 \| 656 \| \| MPN524Fa \| GCTGAACTTAGTTGGCAGCA \| 646107-646126 \| 682 (type 1) \| \| MPN524R \| CCTTGGGCAGATGGTTTTT \| 645445-645463 \| 661 (type 2) \| \| MPN655F \| AGCTTGATCAGTGTGTTGCCTA \| 779964-779985 \|  \| \| MPN655R \| TGCCAACAAAATTGCAAAAA \| 780698-780717 \| 754 \| |
| --- | --- | --- | --- | --- | --- | --- | --- | --- | --- | --- | --- | --- | --- | --- | --- | --- | --- | --- | --- | --- | --- | --- | --- | --- | --- | --- | --- | --- | --- | --- | --- | --- | --- | --- | --- | --- | --- | --- | --- | --- | --- | --- | --- | --- | --- | --- | --- | --- | --- | --- | --- | --- | --- | --- | --- | --- | --- | --- | --- | --- | --- | --- | --- | --- | --- | --- | --- | --- | --- | --- | --- | --- | --- | --- | --- | --- | --- | --- | --- | --- | --- | --- | --- | --- | --- | --- | --- | --- | --- | --- | --- | --- | --- | --- | --- | --- | --- | --- | --- | --- | --- | --- | --- | --- | --- | --- | --- | --- | --- | --- | --- | --- | --- | --- | --- | --- | --- | --- | --- | --- | --- | --- | --- | --- | --- | --- | --- | --- | --- | --- | --- | --- | --- | --- | --- | --- | --- | --- | --- | --- | --- | --- | --- | --- | --- | --- | --- | --- | --- | --- | --- | --- | --- | --- | --- | --- | --- | --- | --- | --- | --- | --- | --- | --- |

^a^ Same forward primer paired with gene-specific reverse primers was used to amplify MPN094, MPN368 and MPN524 regions. The same primer was used as reverse primer in amplification of MPN465.

^b^ Identical forward primer combined with gene-specific reverse primers was used to amplify MPN151 and MPN287 regions.

For further analysis, individual products were extracted from 1.5% agarose (QIAquick^®^  Gel Extraction Kit, QIAGEN Sciences).
