# Supplementary material for: Mycoplasma pneumoniae Large DNA Repetitive Elements RepMP1 Show Type Specific Organization among Strains
Source: PLoS One. 2012 Oct 16;7(10):e47625. doi: 10.1371/journal.pone.0047625 (PMC3472980; doi:10.1371/journal.pone.0047625)
Supplement: Table S2 — RepMP1 and DUF16-containing genes and their position in M. pneumoniae M129 genome. (DOCX) [file pone.0047625.s007.docx]

Table S2: RepMP1 and DUF16-containing genes and their position in *M. pneumoniae* M129 genome.

| **Locus** | **RepMP1-core^a^** |  | **Position within the M129 genome** | **DUF16^b^** |
| --- | --- | --- | --- | --- |
| **MPN037** | + | hypothetical protein | 45770-46213 | - |
| **MPN094** | + | hypothetical protein | 116287-116709 | + |
| **MPN100** | + | hypothetical protein | 129458-130009 | + |
| **MPN127** | + | hypothetical protein | 164484-165026 | + |
| **MPN130^c^** | + | hypothetical protein | 169042-169464 | + |
| **MPN137^c^** | + | hypothetical protein | 178143-177457 | + |
| **MPN138^c^** | + | hypothetical protein | 178892-178392 | + |
| **MPN139** | + | hypothetical protein | 179620-179129 | + |
| **MPN151** | + | hypothetical protein | 199139-199540 | + |
| **MPN204** | + | hypothetical protein | 247655-248101 | + |
| **MPN283** | + | hypothetical protein | 336479-336826 | + |
| **MPN287** | + | hypothetical protein | 343764-344120 | + |
| **MPN368** | + | hypothetical protein | 439220-439762 | + |
| **MPN410** | + | hypothetical protein | 494694-495140 | + |
| **MPN465** | + | conserved hypothetical protein | 569244-568645 | - |
| **MPN484** | + | hypothetical protein | 588613-588302 | + |
| **MPN501** | + | hypothetical protein | 608167-608757 | + |
| **MPN504** | + | hypothetical protein | 612740-613120 | + |
| **MPN524** | + | hypothetical protein | 646051-645545 | + |
| **MPN655** | + | hypothetical protein | 780008-780622 | + |
|  |  |  |  |  |
| **MPN010** | **-** | hypothetical protein | 12257-12652 | + |
| **MPN013** | **-** | hypothetical protein | 14992-15765 | + |
| **MPN038** | **-** | hypothetical protein | 46792-46442 | + |
| **MPN104** | **-** | hypothetical protein | 134583-134897 | + |
| **MPN145** | **-** | hypothetical protein | 192214-192753 | + |
| **MPN675** | **-** | hypothetical protein | 799467-799162 | + |

^a^ Four RepMP1-genes (MPN127, MPN151, MPN287 and MPN504) were added after BLAST analysis when compared with previous data [5].

^b^ Coiled coil domain was identified within C-terminal region of these proteins [25].

^c^ Genes involved in recombination [5].
